# Magnesium uptake by connecting fluid-phase endocytosis to an intracellular inorganic cation filter

**DOI:** 10.1038/s41467-017-01930-5

**Published:** 2017-12-01

**Authors:** Sandra H. Klompmaker, Kid Kohl, Nicolas Fasel, Andreas Mayer

**Affiliations:** Department of Biochemistry, University of Lausanne, Ch. des Boveresses 155, CH-1066 Epalinges, Switzerland

## Abstract

Cells acquire free metals through plasma membrane transporters. But, in natural settings, sequestering agents often render metals inaccessible to transporters, limiting metal bioavailability. Here we identify a pathway for metal acquisition, allowing cells to cope with this situation. Under limited bioavailability of Mg^2+^, yeast cells upregulate fluid-phase endocytosis and transfer solutes from the environment into their vacuole, an acidocalcisome-like compartment loaded with highly concentrated polyphosphate. We propose that this anionic inorganic polymer, which is an avid chelator of Mg^2+^, serves as an immobilized cation filter that accumulates Mg^2+^ inside these organelles. It thus allows the vacuolar exporter Mnr2 to efficiently transfer Mg^2+^ into the cytosol. *Leishmania* parasites also employ acidocalcisomal polyphosphate to multiply in their Mg^2+^-limited habitat, the phagolysosomes of inflammatory macrophages. This suggests that the pathway for metal uptake via endocytosis, acidocalcisomal polyphosphates and export into the cytosol, which we term EAPEC, is conserved.

## Abstract (web summary)

Metal bioavailability is frequently limited by sequestering agents which makes them inaccessible to cells. Here the authors show that cells can increase Mg^2+^ uptake via fluid phase endocytosis and accumulate this metal in their vacuole loaded with polyphosphate, and later can be exported to the cytosol.

## Introduction

Bioavailability of nutrient metals is commonly a limiting factor for microorganisms in their natural environment. This is especially the case for unicellular organisms with limited spatial motility such as yeast. Yeast species are found on plants and in soil^1^, habitats known to be rich in chelating agents like organic acids and tannins, which can limit the fraction of metal ions that are available for uptake across membranes^2–5^. Since plasma membrane metal transporters need free ions for uptake, mitigating strategies for metal acquisition in environments with low metal bioavailability would prove beneficial.

An effective strategy involves uptake of a metal-chelate across the plasma membrane and subsequent release of the metal in the cell. This mechanism has been described for iron and, more recently, zinc. *Saccharomyces cerevisiae* cells can acquire iron through ARN family plasma membrane transporters for siderophores that have been produced by other fungi or bacteria^6–10^. Pra1 was identified in the fungal pathogen *Candida albicans* as a “zincophore”, a secreted protein sequestering zinc from the host cell in order to overcome nutritional immunity during invasive growth^11^. The intracellular fate of the metal-loaded siderophores and the mechanisms of metal release are not well described and depend on the type of siderophore and on the transporter involved. Arn1-mediated uptake of ferrichrome leads to its accumulation in the cytosol, where it showed slow iron release kinetics^12^. Sit1-dependent internalization of ferrioxamine B leads to vacuolar storage after uptake^13^. *C. albicans* and *Leishmania* cells extract iron from host hemoglobin following endocytosis of the compounds and their degradation in lysosomes^14,15^.

Yeast vacuoles are acidocalcisome-like organelles that influence the equilibrium between metal supply and demand and are equipped with a vast number of transporters that load and discharge the organelle of polyphosphate (polyP), divalent metal ions and basic amino acids^16–19^. These features are shared across species with acidocalcisomes, lysosome-related organelles that are conserved from bacteria to man^20^. The cellular functions of acidocalcisomes are poorly understood. Like yeast vacuoles, they are regarded as storage compartments for metal cations and phosphate, which they accumulate to high concentrations^21^. PolyP is a ubiquitous inorganic polymer that is present in all kingdoms of life. It consists of long chains of inorganic phosphate units linked by phosphoric anhydride bonds. PolyP efficiently chelates divalent metal ions^22^ but interacts also with other acidocalcisomal cations such as spermidine and basic amino acids^23,24^. PolyP is implicated in pathogenicity of bacteria and parasites^25,26^ and in blot clotting in mammals^27^. In lower eukaryotes, polyP is synthesized by the polyphosphate polymerase Vtc4, a component of the membrane-anchored vacuolar transporter chaperone (VTC) complex, which consists of the subunits Vtc1 through Vtc5^28,29^. Cells lacking polyP show increased resistance to Ni^2+^, Cd^2+^ and Mn^2+^ stress^30,31^, whereas mutants with increased polyP levels, such as *pho80*Δ, are hypersensitive to high concentrations of Mn^2+^ and Co^2+^ ^32^.

Here we investigate how *Saccharomyces cerevisiae* copes with limited bioavailability of metal ions, mimicking this situation by growing the cells in medium with EDTA. We find that upregulation of fluid-phase endocytosis under these conditions leads to increased transfer of extracellular solutes to the vacuole, where metal ions are accumulated and then exported into the cytosol.

## Results

### PolyP-deficient cells are sensitive to low metal availability

**Fig. 1:**
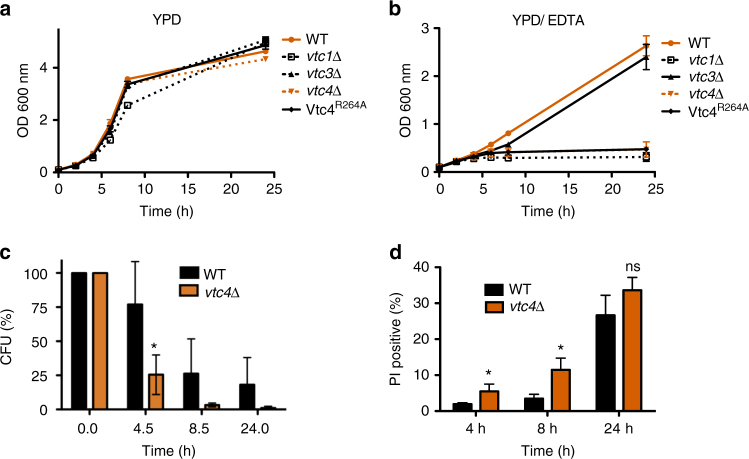
Growth and survival of wild-type and *vtc4*Δ cells. Cells were grown (**a**) in liquid YPD or (**b**) in YPD supplemented with 1 mM EDTA and the OD_600__nm_ was determined at the indicated times. **c** Capacity to form colonies. Cells were grown on YDP/EDTA for the indicated periods of time, cell density was determined, and equal numbers of cells were withdrawn and plated on solid YPD plates. Colony-forming units (CFU) were counted after 2 days of incubation at 30 °C. **d** Live/dead cell staining. Wild-type and *vtc4*Δ cells were collected from YPD/EDTA cultures and stained with propidium iodide (PI) at the indicated time points. The fraction of dead cells in the population was determined by flow cytometry. Differences were evaluated using Student’s *t* test comparing *vtc4*Δ and wild-type values at the respective time points; ns, non-significant; *p ≤ 0.05. The results represent the mean ± SD of 3 (**a**, **b**, **d**) or 5 (**c**) independent experiments

Cells lacking polyP and the vacuolar polyP polymerase VTC show normal growth in standard rich media such as YPD (Fig. 1a). When the bioavailability of divalent metal ions was reduced by addition of the chelator EDTA, wild-type cells only reduced their growth rate (Fig. 1b), whereas cells lacking VTC subunits (*vtc1*Δ*, vtc4*Δ) arrested growth. Cells expressing Vtc4^R264A^, an active-site mutant that allows the assembly of an intact yet inactive VTC complex, displayed the same growth phenotype^28^. *vtc3*Δ cells grew like wild-type because Vtc3 can be substituted for by its homolog Vtc2^28,33^. The vast majority of polyP in *S. cerevisiae* is stored in the vacuole. However, polyP is a cell wall component in a large variety of fungi, and in *S. cerevisiae* evidence for and against a cell wall fraction was presented^34–38^. Since a portion of the VTC complex localizes to the cell periphery^33^, where it could potentially synthesize polyP for transfer into the periplasm, we analyzed the localization of GFP-tagged Vtc4 under metal limitation (Supplementary Fig. 1). Vacuolar localization was confirmed by co-localization with the marker dye FM4-64. On metal-replete YPD, the majority of GFP-Vtc4 localized to the vacuole but a significant portion was found at the cell periphery. On YPD/EDTA, GFP-Vtc4 disappeared from the cell periphery and localized exclusively to the vacuole and vacuole-associated vesicles. This observation argues against a role of the peripheral pool of VTC in metal acquisition and suggests that vacuolar rather than cell wall-associated polyP supports growth under metal limitation.

Cells experiencing starvation conditions may either die or arrest the cell cycle. We distinguished between these possibilities by subjecting cells to different periods of metal starvation on YPD/EDTA, followed by plating on metal-replete YPD medium to measure the number of colony-forming units (CFU) (Fig. 1c). During the first 4 h of adaptation to metal limitation, the number of colony-forming units of wild-type cells dropped to 75% of the initial value, that of *vtc4*Δ cells was reduced to 25%. We tested for the presence of dead cells by propidium iodide (PI) staining and flow cytometry (Fig. 1d). Propidium iodide is excluded from living cells but brightly stains dead cells, which lose metabolic activity that is required to maintain the selective permeability barrier of the plasma membrane. 3% of wild-type cells were PI-positive after 8 h in YPD/EDTA. Only 12% of *vtc4*Δ cells stained with PI at this time point, even though the number of CFUs for this strain had declined by >95% over the same period. Prolonged exposure to EDTA for 24 h killed 25% of wild-type cells, but the survivors retained their capacity to form colonies, which was not the case for *vtc4*Δ cells. Thus, a large fraction of polyP-deficient *vtc4*Δ cells^39^ remain alive and metabolically active in YPD/EDTA, but they rapidly arrest growth. This suggests that polyP is necessary to preserve the capacity of cells to divide on YPD/ETDA.

### PolyP changes under limited metal bioavailability

**Fig. 2:**
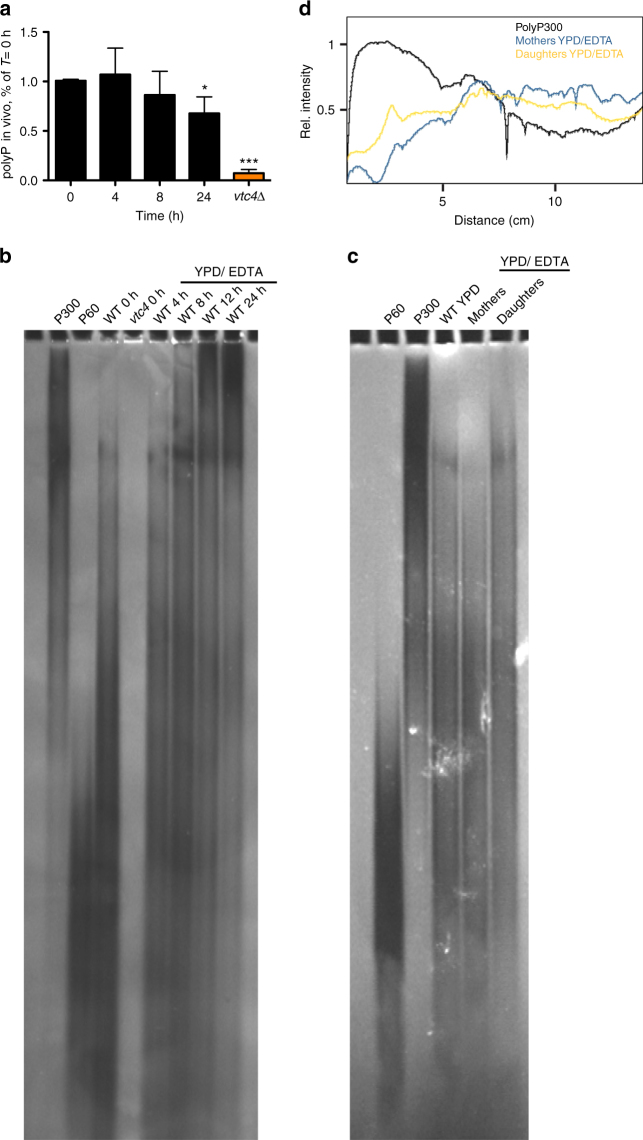
Amounts and chain length of polyP on YPD/EDTA. **a** PolyP content in wild-type cells on YPD/EDTA. Cells were grown on YPD/EDTA for the indicated periods of time, collected, and polyP content was assayed. Results represent mean ± SD of three independent experiments. Statistical differences were determined using Student’s *t* test comparing polyP levels from *T* = 0 h with the later time points and the *vtc4*Δ strain; *p ≤ 0.05; ***p ≤ 0.001. **b** Chain length. PolyP was extracted from cells growing on YPD/EDTA for the indicated periods of time. PolyP was purified from cells by glass bead rupture and phenol/chloroform extraction. Samples were treated with RNAse A and proteinase K before 30 nmol of polyP were resolved on a 15% polyacrylamide gel, followed by negative staining with DAPI. **c** Mother (m) and daughter (d) fractions were separated after 16 h of growth on YPD/EDTA. PolyP was extracted from cells as in (**b**), resolved on a 20% polyacrylamide gel and stained with DAPI. PolyP isolated from a non-separated population of wild-type cells growing logarithmically on YPD is shown for comparison. **d** The signal intensity in the lanes with mother and daughter samples from (**c**) was quantified using ImageJ, starting at the top of the gel. The profile for polyP300 was included for comparison

On medium with limiting amounts of zinc, yeast cells grow linearly instead of exponentially^40^. This is due to the fact that mothers born on rich media continue dividing, whereas newly born daughters arrest growth in the G1 phase of the cell cycle. We observed the same behavior for wild-type yeast cells grown on YPD/EDTA (Supplementary Fig. 2). Given that polyP is crucial for growth on YPD/EDTA, we analyzed changes of polyP under these conditions. Time-course analysis showed that wild-type cells maintained their polyP levels on YPD/EDTA (Fig. 2a). PolyP chain length distribution was examined by gel electrophoresis and negative staining of the gel with DAPI. YPD-grown wild-type cells (*T* = 0 h) contained mostly polyP of 60–100 residues (Fig. 2b). On YPD/EDTA, long-chain polyP of >300 residues increased strongly. After 24 h of growth, the majority of the population consisted of non-dividing daughter cells. These are readily identifiable due to the fact that they carry only a single bud scar, a cell wall remnant from a preceding cell division that can be stained with Calcofluor white. In order to test whether polyP differed in dividing mothers and arrested daughters, we separated these two fractions. The inoculum (mothers) was cross-linked to biotin-fluorescein, washed and cultivated for 16 h on YPD/EDTA. Mothers and daughters were separated by purification with streptavidin-coated, magnetic Dynabeads, which adsorb preferentially biotin-labeled mothers. Fraction purity was assessed microscopically by counting fluorescein-positive and -negative cells. The mother fraction contained 35–45% of daughters, largely due to daughters that had not yet completed cytokinesis and separated from the mothers. Daughter fractions contained <10% mother cells. PolyP from daughter cells contained a higher amount of long-chain polyP, whereas short and intermediate polyP chains dominated in the mother fraction, especially if one takes into consideration that a large part of the long-chain polyP visible in the mother fraction must originate from its inevitable 35% contamination with daughter cells (Fig. 2c). If vacuolar polyP degradation is impaired, vacuoles accumulate extremely long polyP chains of >300 residues^41^. Together with the fact that also the purified catalytic domain of the complex synthesizes very long-chain polyP^28^, this suggests that VTC is a highly processive enzyme. The presence of shorter chains in YPD/EDTA-grown mothers can then be taken as an indication of turnover of polyP by polyphosphatases. Since only the mothers divide on YPD/EDTA, whereas daughters do not, growth on YPD/EDTA coincides with and might require polyP turnover.

### Mg^2+^ limits growth of polyP-deficient cells on YPD/EDTA

**Fig. 3:**
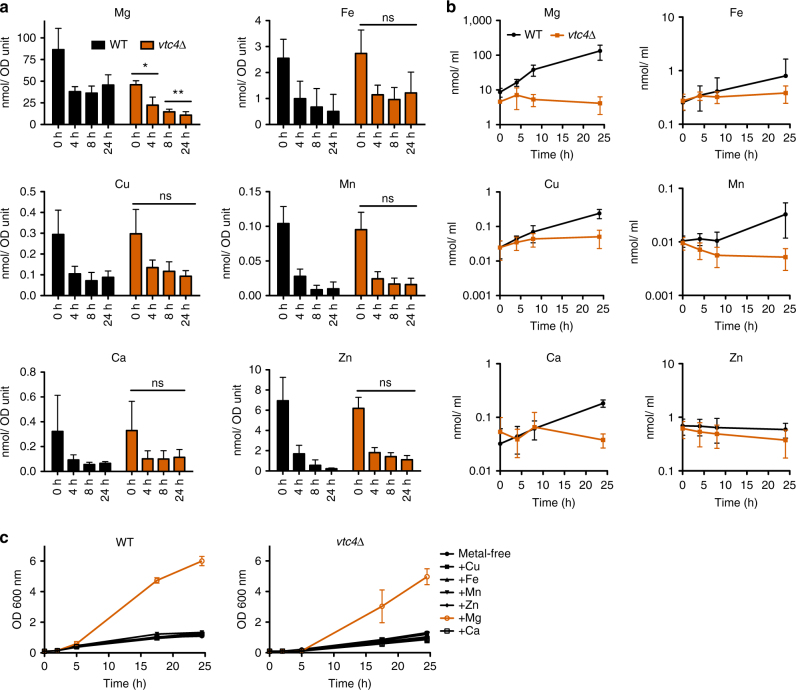
Metal extraction from YPD/EDTA. Time-course ICP-MS analysis. Cells were pre-cultured on YPD and transferred to YPD/EDTA. At the indicated time points, aliquots were withdrawn, cell density was determined, the cells were sedimented and their content of magnesium, iron, zinc, copper, manganese and calcium was measured. **a** Values per OD_600__nm_ unit of cells and (**b**) the values per culture volume, which allows to judge whether there is net transfer of metal from the medium into the accumulating biomass. **c** Growth on YPD/Chelex medium. Wild-type and *vtc4*Δ cells were pre-cultured for 5 h on YPD/EDTA and transferred to YPD/Chelex medium supplemented with the indicated metal to final concentrations that are sufficient to support vigorous continuous growth of non-depleted wild-type cells: 0.25 μM CuSO_4_; 1.2 μM FeCl_3_; 2.6 μM MnSO_4_; 2.5 μM ZnSO_4_; 0.9 mM CaCl_2_; 4.2 mM MgSO_4_. The OD_600nm_ was measured at the indicated time points after transfer. **a**–**c** Results represent mean ± SD of at least three independent experiments. Statistical differences in **a** were determined using Student’s *t* test comparing *vtc4*Δ and wild-type cells at each time point; *p ≤ 0.05; **p ≤ 0.005

Since EDTA chelates a range of divalent metal ions, we asked which metal restricts growth of polyP-deficient strains. Cells were pre-cultured on YPD and then cultivated in YPD/EDTA for different periods of time before metal levels were determined by inductively-coupled plasma (ICP)-MS analysis. The metal content per cell decreased over time for Fe, Zn, and Mn, with no major differences between wild-type and *vtc4*Δ cells (Fig. 3a). This suggests that cells grew by using, at least partially, stores of these metals that are independent of polyP. Wild-type cells stabilized the levels of Mg, Cu, and Ca at 35–50% of the initial value (Fig. 3a). *vtc4*Δ cells showed similar stabilization for Cu and Ca, but their content of Mg kept decreasing to <10% over 24 h (Fig. 3a). Even on YPD without EDTA (*T* = 0 min), *vtc4*Δ cells had 40% less Mg than wild-type cells. This suggests that wild-type cells extract enough Mg, Ca, and Cu from YPD/EDTA to allow them to stabilize their content of these three ions. However, the stabilization of Mg levels depends on polyP.

This picture was corroborated by integrating the metal content over all the cells in the culture, which allows to assay the net transfer of metals from the medium into the sedimentable, living material. Whereas cell-associated Mg and Ca increased in the pellets of wild-type cultures more than 20-fold over 24 h on YPD/EDTA (Fig. 3b), other metals showed no (Zn) or only moderate increases of <5-fold (Mn, Cu, Fe). Ca^2+^ levels reproducibly spiked after 4 h (Fig. 3b), which might be due to activation of the calcineurin/ Crz1 pathway, which strongly augments Ca^2+^ uptake^42^. Apart from a moderate increase in Cu, *vtc4*Δ cultures did not accumulate any of the tested metals, which reflects their lack of growth on EDTA.

On the basis of these results, we hypothesized that EDTA might limit growth of cells by reducing the bioavailability of Mg^2+^. In order to test this, we depleted cellular metal pools by incubating the cells in YPD/EDTA for 5 h (Fig. 3c). Subsequently, cells were diluted into YPD that had been depleted of metals by chromatography over a Chelex ion-exchange matrix, which reduced the Mg content of this medium to 10 µM (determined by ICP-MS). This medium was then supplemented with a single metal at a time. Only addition of Mg^2+^ allowed cells to proliferate and the response was similar for wild-type and *vtc4*Δ cells. This suggests that cells grown on YPD/EDTA do not maintain a sufficiently large stock of Mg^2+^ to support further cell divisions under Mg^2+^ limitation. Therefore, their growth on YPD/EDTA depends on continuous extraction of Mg^2+^ from this medium.

### A vacuolar Mg^2+^ exporter is required for Mg^2+^ uptake

Several intracellular pools of Mg^2+^ exist, with transporters and channels controlling the fluxes between them. Alr1 mediates the majority of Mg^2+^ uptake at the plasma membrane^43^. Cells lacking Alr1 depend on supplementation of their media with high concentrations of Mg^2+^ for wild-type-like growth. Overexpression of its homolog Alr2 can rescue this phenotype^43,44^. The Mrs2/ Mfm1 complex controls Mg^2+^ import into mitochondria^45,46^ and Mme1 exports Mg^2+^ from this organelle^47^. A vacuolar Mg^2+^ importer has not been identified, but Mnr2 mediates efflux of Mg^2+^ from vacuoles^48^.

**Fig. 4:**
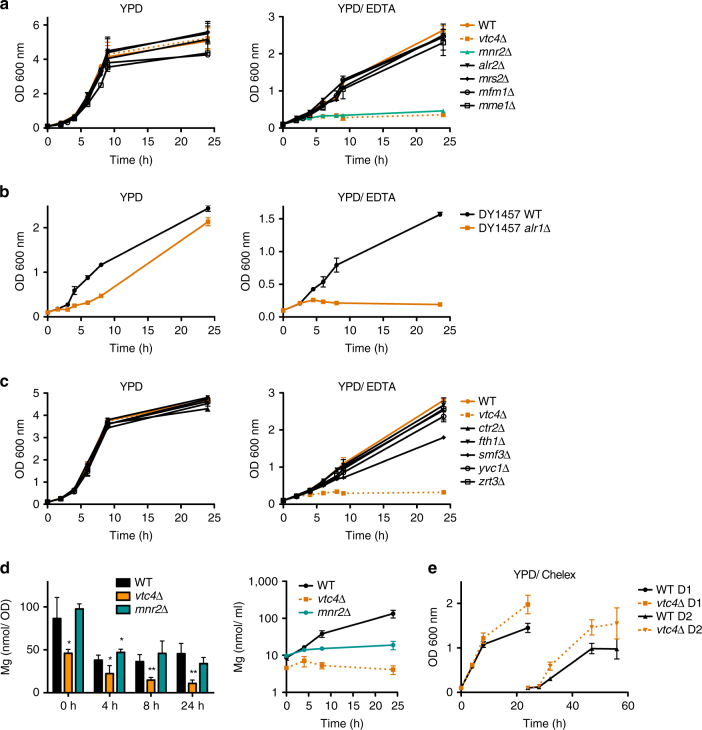
Role of metal transporters for growth on YPD/EDTA. **a** Magnesium transporter mutants were pre-cultured on YPD, transferred to YPD or YPD/EDTA, and the OD_600__nm_ was measured at the indicated times after transfer. **b** Growth of *alr1*Δ cells was assayed as in (**a**). **c** Growth of vacuolar metal exporter mutants was assayed as in (**a**). **d** Time course ICP-MS analysis of the magnesium content of *mnr2*Δ cells at the indicated times after transfer from YPD to YPD/EDTA. Values per cell and per culture volume are shown. Note that the values for wild-type and *vtc4*Δ are the same as in Fig. 3b because *mnr2*Δ cells had been tested as part of the same experiments. **e** Growth of wild-type and *vtc4*Δ cells on Chelex-treated YPD. Cells were pre-cultivated in YPD, washed and transferred to YPD/Chelex. The OD_600__nm_ was followed for one day (D1). The cells were then re-inoculated in fresh YPD/Chelex and growth was followed for another day (D2). The results represent mean ± SD of at least three independent experiments. Differences in **d** were evaluated using Student’s *t* test comparing *vtc4*Δ and *mnr2*Δ cells with wild-type cells at each time point; *p ≤ 0.05; **p ≤ 0.01

In order to test whether a particular intracellular Mg^2+^ pool is essential for cells to grow on metal-limiting medium, we assayed knockout strains for these Mg^2+^ transporters for growth on YPD/EDTA (Fig. 4a, b). Cells lacking the plasma membrane transporter Alr1 grew only slowly on YPD and did not grow on YPD/EDTA (Fig. 4b). *Alr2*Δ, *mrs2*Δ, *mfm1*Δ, and *mme1*Δ strains grew like wild-type cells on both media. Interestingly, *mnr2*Δ cells mimicked the phenotype of a *vtc4*Δ strain. They rapidly ceased growth on YPD/EDTA but grew like wild-type on YPD (Fig. 4a). By contrast, mutants for other known vacuolar metal exporters such as z*rt3*Δ (Zn^2+^), y*vc1*Δ (Ca^2+^), *ctr2*Δ (Cu^2+^), and *fth1*Δ (Fe^2+^) grew like wild-type (Fig. 4c). Only s*mf3*Δ (Fe^2+^) cells showed slightly reduced growth kinetics. This suggests that growth on YPD/EDTA specifically requires the vacuolar Mg^2+^ exporter Mnr2. Furthermore, ICP-MS analysis showed that, similarly as *vtc4*Δ cells, *mnr2*Δ cells did not efficiently accumulate Mg from the growth medium (Fig. 4d, right panel). However, *mnr2*Δ cells did not show the decrease in Mg content that was observed in *vtc4*Δ cells, showing that *mnr2*Δ cells could not mobilize their vacuolar Mg when needed (Fig. 4d, left panel).

These results suggest that both uptake of Mg^2+^ through the plasma membrane and mobilization of Mg^2+^ from vacuoles contribute to growth on YPD/EDTA. This is in line with the observation that *vtc4*Δ cells grow even slightly better than wild-type on Chelex-treated medium, which is low in Mg^2+^, but in which this Mg^2+^ is fully bioavailable and thus accessible to the plasma membrane transporters Alr1 and Alr2 (Fig. 4e). Under these conditions, plasma membrane uptake can apparently satisfy the Mg^2+^ requirements for growth. By contrast, growth under conditions of limited bioavailability requires vacuolar polyP and vacuolar Mg^2+^ export.

### Mg^2+^ uptake requires fluid-phase endocytosis and retrograde traffic

**Fig. 5:**
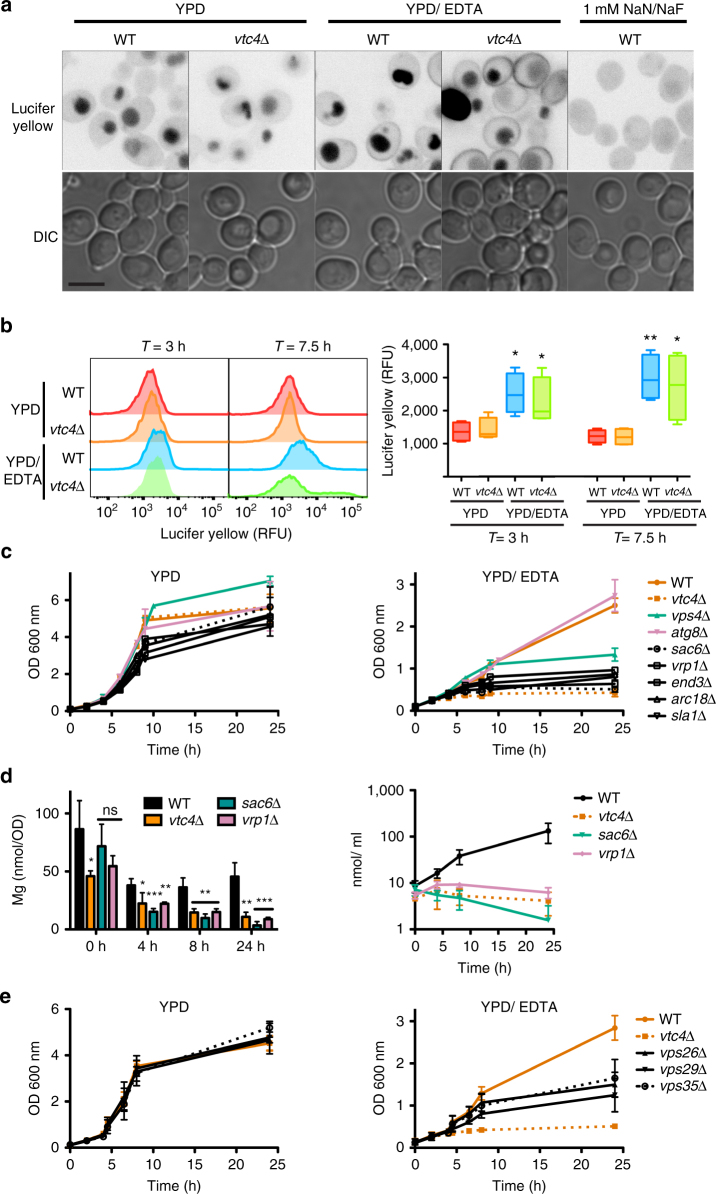
Metal limitation and endocytosis. **a** Lucifer yellow uptake assayed by microscopy. Cells were pre-cultured on YPD, then grown on YPD or YPD/EDTA for 2 h, sedimented and resuspended in the same medium containing 2.3 mM Lucifer yellow. After 75 min of incubation, the cells were washed 5 times with PBS and analyzed by fluorescence and differential interference contrast (DIC) microscopy. Cells depleted for ATP by NaN_3_/ NaF treatment (1 mM each) were used as a negative control. Scale bar: 5 μm. Gray values of the fluorescence images have been inverted. **b** Lucifer yellow uptake quantified by flow cytometry. Cells were incubated with Lucifer yellow and washed as in (**a**). Their fluorescence was analyzed by flow cytometry. The left panel shows a representative histogram. The right panel shows Lucifer yellow content from four independent experiments. Whiskers represent minimum and maximum of all of the data, the bottom and the top of the box show first and third quartiles and the band inside the box the median. **c** Growth analysis of membrane trafficking mutants on YPD and YPD/EDTA was performed as in Fig. 4a. **d** ICP-MS analysis of Mg content in the endocytosis mutants *vrp1*Δ and *sac6*Δ, grown on YPD/EDTA, was performed as in Fig. 3a. Values per cell and per culture volume are shown. **e** Growth analysis of retromer complex mutants on YPD and YPD/EDTA was performed as in Fig. 4a. **b**–**e** The results represent mean ± SD of at least three independent experiments. Differences were evaluated using Student’s *t* test comparing (**b**) wild-type and *vtc4*Δ cells ± 1 mM EDTA at each time point, and (**d**) *vtc4*Δ and *mnr2*Δ cells with wild-type at each time point; ns, non-significant, *p ≤ 0.05; **p ≤ 0.01; *** *p* ≤ 0.005

Given that polyP, which is enclosed in vacuoles, facilitated Mg^2+^ uptake into the cytosol, we explored how Mg^2+^ could be brought in contact with polyP and tested fluid-phase endocytosis as a possibility. We quantified this process using the fluorescent fluid-phase endocytosis marker Lucifer yellow^49^. Cells were grown on YPD and YPD/EDTA for 2 and 6.5 h before adding Lucifer yellow and incubating them for an additional hour. The cells were sedimented and washed. Dye uptake was analyzed qualitatively by fluorescence microscopy (Fig. 5a) and quantified by flow cytometry (Fig. 5b). Both wild-type and *vtc4*Δ cells showed significantly higher Lucifer yellow fluorescence after 3 h of growth in YPD/EDTA when compared to YPD. This difference increased further after 7.5 h on YPD/EDTA, suggesting that the cells had upregulated their capacity for fluid-phase endocytosis as a consequence of limited metal availability.

We tested the growth effects of mutations perturbing fluid-phase endocytosis (*sac6*Δ*, vrp1*Δ*, end3*Δ*, arc18*Δ*, sla1*Δ), vacuolar protein sorting at the endosome (*vps4*Δ), and autophagy (*atg8*Δ, Fig. 5c). Wild-type and *atg8*Δ cells showed robust growth during 24 h of incubation on YPD/EDTA. In contrast, cells lacking components of the endocytic machinery mimicked the growth defect of polyP-deficient cells. *vps4*Δ cells displayed an intermediate growth phenotype. ICP-MS analysis of the endocytosis mutants *sac6*Δ and *vrp1*Δ confirmed their defects in Mg^2+^ acquisition (Fig. 5d). Continuous uptake of Mg^2+^ should necessitate a compensatory fluid exit from vacuoles in order to allow extrusion of non-used compounds that had been taken up through fluid-phase endocytosis. In line with this, mutants in the Retromer complex (*vps35*Δ*, vps29*Δ*, vps26*Δ), which mediates vesicular exit from endosomes and vacuoles^50–52^, showed similar growth defects on EDTA as the endocytosis mutants (Fig. 5e). Taken together, these observations support a model in which limited bioavailability of Mg^2+^ prompts cells to upregulate fluid-phase endocytosis in order to deliver extracellular ions into the vacuole. The polyP that is accumulated in this compartment is a strong chelator for Mg^2+^ ^22,23^, which probably enables the cell to retain the endocytosed Mg^2+^. Finally, Mg^2+^ is transferred from the vacuolar lumen to the cytosol via the dedicated Mg^2+^ exporter Mnr2.

### PolyP is needed in Mg^2+^-poor environments across species

**Fig. 6:**
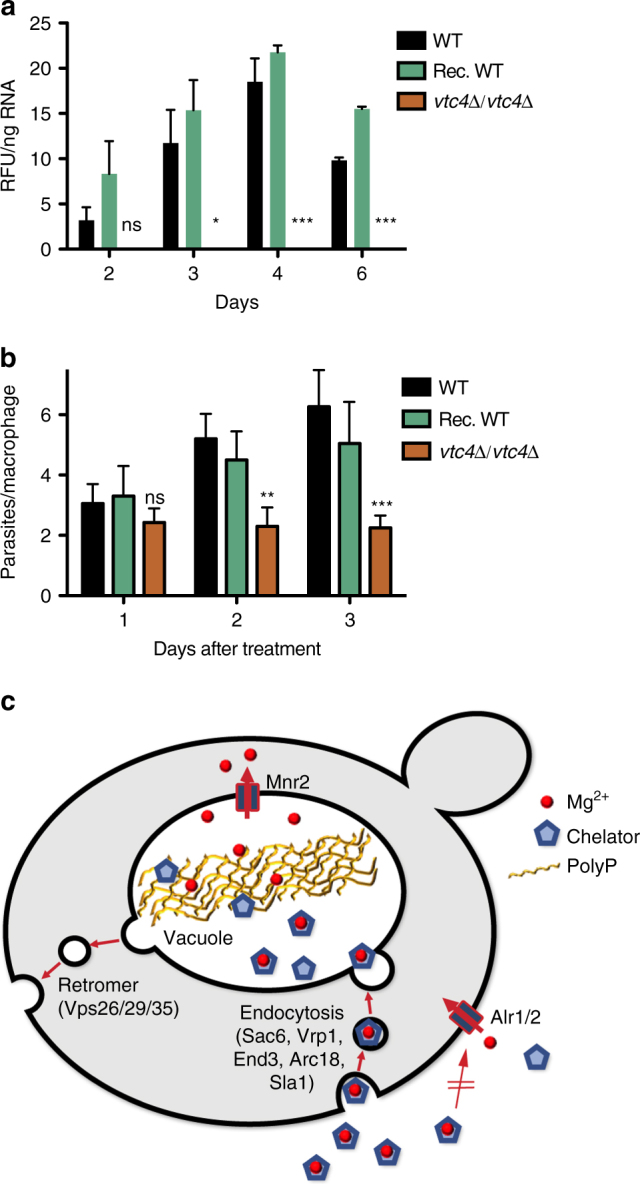
PolyP and growth of *Leishmania major* in macrophages. **a** PolyP levels during promastigote growth. Promastigotes of wild-type, a double-knockout of LmVTC4 (*vtc4*Δ/*vtc4*Δ) and of the double-knockout reconstituted with a wild-type allele of LmVTC4 (rec. WT) were inoculated in M199 medium. They were analyzed for their polyP content at the indicated times after inoculation. Day 4 corresponds to logarithmic growth, day 6 to stationary phase. **b** Proliferation inside macrophages. Bone-marrow-derived macrophages were infected with late stationary phase promastigotes. Microscopy pictures were taken 24, 48, and 72 h after infection and the number of amastigotes was counted in >100 macrophages per cell line. **a**, **b** The results represent mean ± SD. Statistical differences were evaluated using Student’s *t* test, comparing *vtc4*Δ*/vtc4*Δ with wild-type cells at each time point from 4 independent experiments: ns, non-significant; *p ≤ 0.05; **p ≤ 0.005; ***p ≤ 0.0005. **c** Working model of the EAPEC pathway. When cells cannot acquire sufficient amounts of free Mg^2+^ through their plasma membrane transporters (Alr1/2), they endocytose bulk fluid phase with chelated Mg^2+^ and transfer this solution into the acidocalcisome-like vacuole. Here, high concentrations of polyP, in combination with acidic pH, favor liberation of the metal ions from chelating substances and their adsorption to polyP. This leads to the enrichment of Mg^2+^ inside vacuoles and facilitates its transport into the cytosol via the Mg^2+^ exporter Mnr2

We sought to test whether polyP in acidocalcisome-like organelles might act similarly in a more physiological setting and whether its requirement for Mg^2+^ acquisition might be conserved in evolution. To this end, we utilized macrophages, which phagocytose pathogens and finally degrade them. Some pathogens, however, evade or resist digestion and even multiply within the phagolysosomes, subverting the host cell machinery to proliferate. Access to nutrients and specifically to Mg^2+^ is limited within the phagosome^53–56^. We utilized the trypanosomatid parasite *Leishmania major* as a model. Trypanosomatids contain acidocalcisomes that share polyP and many transporters with yeast vacuoles^57^. After entering the mammalian host, *Leishmania* is taken up by macrophages, where it resides and proliferates in the phagolysosome^58^. Growth in these phagolysosomes is limited by Mg^2+^ ^59^. We tested for a potential role of polyP by creating a homozygous deletion mutant of *L. major* VTC4 and a rescued version of this cell line (VTC4 2 S), in which a functional VTC4 allele was re-integrated into the genome of the homozygous deletion mutant^60^ (Supplementary Figs. 3 and 4). In order to assess polyP synthesis, we cultivated the promastigote form of the parasites and quantified their polyP using the fluorescent indicator DAPI, which generates a characteristic emission at 550 nm (Fig. 6a). PolyP of wild-type and VTC4 2S cells peaked during logarithmic growth, whereas no polyP was detected in *vtc4*Δ/*vtc4*Δ cells. Equivalent results were obtained by assaying polyP through enzymatic hydrolysis and quantification of the released P_i_ (Supplementary Fig. 5). Next, we infected bone marrow-derived mouse macrophages with these strains for 6 h. Non-phagocytosed parasites were washed away and the number of *Leishmania* cells per macrophage was counted 24, 48, and 72 h after infection (Fig. 6b). The rate of phagocytosis was similar for all cell lines, illustrated by a similar parasite load of 2–3 parasites per macrophage after 24 h. However, the number of *vtc4*Δ/*vtc4*Δ parasites per macrophage did not increase over the next two days, whereas wild-type and VTC4 2S cell numbers doubled in the phagolysosomes and reached a concentration of 5–7 parasites per macrophage. Thus, polyP is necessary for intracellular replication of *Leishmania* in the Mg^2+^-poor environment of the phagolysosome.

## Discussion

Our data identify a novel pathway for acquiring Mg^2+^ by fluid-phase endocytosis. Under limited metal bioavailability, the cells upregulate endocytosis in order to bring soluble metals into their acidocalcisome-like, polyP-loaded vacuole. Our results suggest that uptake via this EAPEC pathway and plasma membrane transport are not mutually exclusive but rather complementary. When Mg^2+^ is scarce but fully bioavailable, a condition that we mimicked through Mg^2+^-depleted but EDTA-free medium, the high-affinity Mg^2+^-transporters in the plasma membrane suffice to support growth. The endocytosis/polyP-dependent EAPEC route becomes essential for growth when bioavailability is low, enabling the cell to cope with this condition, which is often encountered in natural settings^5^.

PolyP avidly binds numerous divalent metal ions and readily forms gels with them^23^. PolyP accumulates in acidocalcisome-like vacuoles to concentrations that can reach hundreds of millimolar of phosphate units^20^. Due to this very high concentration, we expect polyP to outcompete the chelating compounds that limited the bioavailability of Mg^2+^ in the surrounding medium (Fig. 6c). The acidic pH of acidocalcisomes, which is around 5, and their high concentrations of basic amino acids and polyamines, should favor this competition. PolyP should hence sequester Mg^2+^ from the endocytosed fluid phase and concentrate it inside these organelles. Concentration is expected to facilitate its transfer to the vacuolar Mg^2+^ exporter, which then makes the metal available to the cytosol. We consider polyP essentially as an intracellular ion filter that extracts metal ions from a continuous flow of media through the vacuole, which is established by vesicular traffic to and from this organelle. This sets it apart from other strategies of metal acquisition such as siderophores, which are secreted into the extracellular space and then recovered by high-affinity transporters in the plasma membrane^61^.

An interesting open question is how Mg^2+^ that is accumulated through polyP inside vacuoles could become available for the vacuolar Mg^2+^-exporter. Several properties of polyP might be relevant for this, giving rise to the following hypotheses, which are not mutually exclusive: First, polyP is a highly charged polymer that can reach 50–100 kDa and form gels. It binds Mg^2+^ and other metal ions. Its confinement inside acidocalcisome-like vacuoles, which are connected to the environment via vesicular import and export, is expected to create a Donnan equilibrium. Donnan equilibria are established when macromolecules with an affinity for small solutes are confined in a space that is connected to a solute reservoir through a semipermeable barrier, which allows solutes to pass but retains the macromolecules^62^. In the case of the yeast cell, the confinement of polyP inside the vacuole, in combination with vesicular fluid-phase transport to and from this compartment, establishes the conditions for Donnan equilibria. The resulting elevated concentration of free Mg^2+^ in the vacuolar lumen would allow the vacuolar Mg^2+^ exporter to function more efficiently. Second, the vacuolar Mg^2+^ exporter might interact with polyP and take over bound Mg^2+^ directly. Third, polyP might be degraded inside vacuoles in order to liberate bound Mg^2+^. In line with this, mother cells, which continue to grow under limited metal availability and hence keep acquiring Mg^2+^, show shorter polyP fragments than the arrested daughters. If enzymatic hydrolysis of polyP occurred close to the vacuolar membrane, it might liberate Mg^2+^, preferentially for export. Unfortunately, the polyP degradation machinery of yeast vacuoles is poorly explored. Vacuoles contain at least three polyphosphatase activities. So far, only the membrane-associated Ppn2^63,64^ and the soluble Ppn1 could be identified^63^, and their simultaneous deletion did not impair growth on YPD/EDTA (not shown). Fourth, polyP readily associates with a variety of abundant cations. Arg, Lys, K^+^, and polyamines accumulate in vacuoles also in concentrations of tens to hundreds of millimolar, maintained by dedicated vacuolar importers^65,66^. They might compete with Mg^2+^ for binding polyP, facilitating Mg^2+^ release. Fifth, polyP forms gel phases that separate adsorbed molecules from the rest of the solution. These phase transitions depend on pH, ionic strength and the type of metal ions bound^67^. Continuous active vacuolar import of counter-ions could maintain their concentrations higher in proximity to their importers, which might promote preferential liberation of Mg^2+^ close to the vacuolar membrane and the vacuolar Mg^2+^ exporter. At this point, these hypotheses cannot be tested because we lack techniques to assess the distributions of short- and long-chain polyP and of the mentioned organic and inorganic cations within vacuoles with sufficient spatial resolution.

Cells might employ metal acquisition via endocytosis and polyP also for acquiring metals other than Mg^2+^. We created experimental conditions that rendered Mg^2+^ limiting, permitting an analysis focused on this metal. However, fluid-phase endocytosis is non-selective and polyP can sequester a broad range of divalent and trivalent metal ions^22^. Vacuoles and acidocalcisomes also contain exporters for transferring a variety of metal ions into the cytosol. Therefore, it is possible that the same pathway and strategy that we identified here for Mg^2+^ might also be used to overcome limited availability of other metal ions. Furthermore, the EAPEC pathway might be conserved among different species because a key feature required for it, an acidocalcisome-like organelle rich in polyP and equipped with metal exporters, is found in all kingdoms of life^20^. This is supported by our results on the proliferation of *Leishmania* in phagolysosomes of inflammatory macrophages. Proliferation of *Leishmania* in phagolysosomes is limited by Mg^2+^ ^59^ and depends on polyP. While during growth in rich media acidocalcisomes are not connected to endocytic traffic^68–71^, such a connection can be established in specific metabolic situations, which allows *Leishmania* acidocalcisomes to accumulate endocytic tracers^72^. Therefore, Leishmania might be able to induce the EAPEC pathway when proliferating inside macrophages. Also, *Trypanosoma brucei*, which causes sleeping sickness, depends on the polyP of its acidocalcisomes for virulence^26^, although in this case, it has not yet been tested whether metal acquisition contributes to the phenotype.

In sum, whereas acidocalcisome-like organelles have frequently been considered as simple storage compartments, our results ascribe to these organelles an active role in metal acquisition for the cytosol. They identify a novel pathway for metal acquisition that utilizes an inorganic intracellular polymer, polyP, as a crucial element. This inorganic polymer is constrained in a specialized organelle and connected to the environment through a flow of aqueous phase generated by endocytosis and vesicular retrograde traffic. It can hence be considered as an intracellular ion filter that is dedicated to extracting Mg^2+^, and perhaps other metal ions, from the environment, and facilitates their enrichment and transfer to the cytosol under conditions of limited bioavailability.

## Methods

### Yeast strains and media

*S. cerevisiae* strains used in this study are listed in Supplementary Table 1. Knockout strains obtained from deletion collections were re-tested by PCR. Primer sequences are listed in Supplementary Table 2. Cells were cultivated overnight at 30 °C in YPD (1% yeast extract, 2% peptone, 2% glucose) to mid-exponential growth phase and inoculated in YPD supplemented with 1 mM EDTA at 0.1 OD units ml^−1^ (corresponds to 0.4×10^7^ cells ml). Since their ion content can vary moderately, new batches of yeast extract and peptone were routinely tested for the induction of a growth defect at an EDTA concentration of 1 mM. Metal-depleted YPD (YPD/Chelex) was prepared by incubating 5 g of Chelex-100 resin (Bio-Rad) per 100 ml YPD overnight on a rotating wheel at 4 °C. The resin was removed by filtration through a 0.2 μm filter and the pH was adjusted to 7 with 1 N HCl. Residual metal concentrations of YPD/Chelex were determined by ICP-MS and are listed in Supplementary Table 3.

### Parasite cell lines and media

*L. major* MRHO/IR/75/ER was used in this study. Promastigotes were grown at 26 °C in M199 medium (Invitrogen AG) complemented with 10% heat-inactivated fetal bovine serum (FBS, Seromed), 50 U ml^−1^ penicillin/streptomycin (Animed), 40 mM Hepes (Animed), 0.6 mg l^−1^ biopterin (Sigma) and 5 mg l^−1^ hemin (Sigma).

### Constructs for LmVtc4 mutant cell lines

To target both *LmjVTC4* alleles in the knockout cell line, plasmids based on the pX63-HYG vector^73^ were used. 787 bp of the VTC4 3′-UTR were amplified from genomic DNA and cloned into the vector using *SmaI* and *BglII* restriction sites. 460 bp of the 5′-UTR were amplified from genomic DNA and cloned into the *HindIII* and *SalI* sites to create pLmjVTC4.k.o.-HYG. To generate pLmjVTC4.k.o.-PAC, the *PAC* gene was released by *SpeI*/ *BamHI* digestion from a modified pX63-PAC vector and used to replace the *HYG* gene in pLmjVTC4.k.o.-HYG^74^. Both plasmids were digested with *BglII*/ *HindII* and the knockout cassettes gel-purified (Promega) and used for parasite transfection. Gene replacements were verified by PCR and Southern blotting.

VTC4 was genomically integrated and re-expressed in the *L. major vtc4*Δ/*vtc4*Δ cell line by replacing the *HYG* resistance gene of the pSSU-int construct^60^ with *NEO* (from pX63-NEO) using *SpeI* and *XbaI*. Lmj*VTC4* was amplified from genomic DNA and cloned into the vector using *ClaI* and *XmaI*. The intergenic region of cysteine proteinase B (CPB) 2.8 was cloned in-between Lmj*VTC4* and *NEO* using *XmaI*/ *SpeI*. The transfection cassette of pSSU-LmjVTC4-NEO was excised by *PacI*/ *PmeI* digestion, gel-purified and used for parasite transfection. Gene integration was verified by PCR and protein synthesis by Western blotting.

### *Leishmania* stable transfection

A concentration of 8–12 μg linear DNA in 5 μl H_2_0 was used to transfect 2 × 10^7^ parasites. Parasites were centrifuged and washed with phosphate buffered saline (PBS). The pellet was resuspended in 100 μl T-cell Nucleofection solution of the Amaxa human T-cell Nucleofector kit (Lonza) and the DNA was added. This mixture was transferred to the Amaxa cuvette and electroporated with the Nucleofector II device, program U-033. Cells were incubated at 26 °C in 4 ml complete M199 medium overnight. After 24 h, cells were plated on semi-solid M199 media containing the appropriate drug to select clonal lines. Drug concentrations were 100 μg ml^−1^ hygromycin, 50 μg ml^−1^ puromycin and 25 μg ml^−1^ neomycin. Colonies were recovered from the plates after 10–15 days.

### Cell viability assays

To determine the number of colony-forming units (CFU), strains were cultured overnight in YPD to mid-exponential growth phase and transferred to YPD with 1 mM EDTA. 0.25 OD_600__nm_ units were collected, pelleted and re-suspended in 250 μl H_2_O. A 10x dilution series was prepared and spread on YPD plates. Colonies were counted after 2 days of incubation at 30 °C. Dead cells were quantified by propidium iodide (PI) staining. 0.25 OD_600__nm_ units were withdrawn from an YPD/EDTA culture. Cells were washed in 1× PBS and 3 μl PI (1 mg ml^−1^) was added to 1 ml of cell suspension in PBS. The suspension was incubated for 20 min in the dark at room temperature. Samples were cooled on ice and flow cytometry was performed within 1 h. Fluorescence from 20,000 cells was acquired on a BD Accuri C6 flow cytometer. PI was excited at 488 nm and the fluorescence detected in the FL3 channel.

### Bud scar analysis

A concentration of 0.6 OD_600__nm_ units were taken from a YPD culture in early logarithmic growth phase and cells were washed twice with 1x PBS/2% glucose. The pellet was resuspended in 500 μl 1× PBS/2% glucose containing 2 mg EZ-Link-Sulfo-NHS-LC-LC-Biotin (Invitrogen) and incubated for 30 min at 30 °C. Cells were washed 3 times with 1× PBS/2% glucose and resuspended in 480 μl 1× PBS/2% glucose. 20 μl of a 5 mg ml^−1^ fluorescein-conjugated-avidin solution (Thermo Fisher Scientific) was added and the suspension was incubated for 10 min at room temperature in the dark. Cells were washed 3 times with 1× PBS/2% glucose and transferred to YPD/EDTA. After 16 h, 1 OD_600__nm_ unit of cells was pelleted and resuspended in 0.9 ml H_2_O. 100 μl of a 1 mg ml^−1^ Calcoflour white stock were added and incubated for 10 min at room temperature. Cells were washed 5 times with water and z-stacks were taken on a confocal spinning disc microscope.

### Metal analysis

Culture volumes of 20 ml were collected by centrifugation (5 min, 3,200× *g*, 4 °C) and cells were washed in TE buffer (10 mM Tris-HCl, 1 mM EDTA, pH 8.0). The pellets were digested in screw-cap tubes overnight in 1 ml 20% nitric acid at 95 °C. Debris was removed in two centrifugation steps (10 min, 16,000 × *g*) in a table top centrifuge and the supernatant was transferred to a fresh tube. ICP-MS analysis was performed with an ELAN DRC II from Perkin Elmer.

### PolyP extraction, quantification and gel electrophoresis

PolyP extraction from yeast and Leishmania for gel analysis was carried out as follows^75^. Two OD_600__nm_ units were collected by centrifugation and the pellet was re-suspended in 250 μl of LETS buffer (10 mM Tris pH 8.0, 0.1 M LiCl, 10 mM EDTA, 0.5% SDS). 250 μl acid phenol (pH 5.2) was added and the suspension transferred to an Eppendorf tube containing 250 μl glass beads. The tubes were vortexed for 5 min at 4 °C and centrifuged (15000× *g*, 5 min, 4 °C). 200 μl of the aqueous phase was transferred to a phase lock gel tube (5 Prime) and extracted with an equal volume of CHCl_3_. The extracts were centrifuged (3 min, 13,000 × *g*, 4 °C) and the upper phase transferred to a fresh tube. PolyP and RNA were precipitated by adding 2.5 volumes of ice-cold ethanol and centrifuged (15 min, 21,000 × *g*, 4 °C). The supernatant was discarded and the pellet washed once in 300 μl ice-cold 75% ethanol. The pellet was dissolved in 25 μl H_2_O after air-drying for 2 h.

Quantification of this polyP was either carried out by Ppx1-mediated hydrolysis and malachite green assay^76^, or by fluorescence spectroscopy with DAPI: A 10× dilution of the polyP extract was prepared in 20 mM Hepes (pH 6.8), 150 mM KCl buffer and further diluted by factors 50 and 100 in a black 96-well plate in buffer containing 20 mM HEPES (pH 6.8), 150 mM KCl and 10 μM DAPI. The plate was equilibrated for 10 min at room temperature. DAPI-PolyP fluorescence was acquired for 20 min at 420 nm excitation and 550 nm emission in a Gemini EM Fluorescence Microplate Reader (Molecular Devices). A standard curve was prepared with PolyP60. For separation of polyP chains by electrophoresis, 15–30 nmol polyP were treated with RNAse, DNAseI and (where indicated) with recombinantly expressed and purified ScPpx1^38^ before samples were resolved on a polyacrylamide gel (acrylamide/bisacrylamide 19:1, Serva) in TBE. Gels were run overnight at 3 mA and 4 °C until the loading dye (30% glycerol, 0.5% bromophenol blue, 1 mM EDTA) front migrated to ½ to ¾ of the gel. Gels were stained in buffer with DAPI (1.5 g l^−1^ Tris base, 2% glycerol, 7.2 μM DAPI) for 45–60 min and destained in the same buffer without DAPI for 30–45 min. Gels were exposed on a UV transilluminator to induce photobleaching, after which pictures of the gel were taken.

PolyP quantification for time-course analysis of *L. major* promastigotes was performed as follows^77^. Cells were pelleted and washed with 50 mM HEPES (pH 7.5). The pellet was resuspended in 400 μl DAPI buffer (20 mM HEPES pH 6.8, 150 mM KCl, 10 μM DAPI) and freeze-thawed by 5 successive passages of 2 min between liquid nitrogen and room temperature. After centrifugation, the pellet was diluted in DAPI buffer in a final volume of 200 μl in a 96-well plate in duplicate and incubated for 10 min at room temperature. Fluorescence was measured in a Gemini EM Fluorescence Microplate Reader (Molecular Devices) at *λ*_ex_ = 420 nm and *λ*_em_ = 550 nm. Values were normalized using co-isolated RNA and corrected for background signal using the polyP-deficient *vtc4*Δ/*vtc4*Δ cell line.

### Microscopy

Images were taken with an UltraView Vox spinning disk confocal microscope (Perkin Elmer-Cetus) connected to an inverted microscope (Carl Zeiss) with a 100× oil immersion objective with a numerical aperture of 1.41 and a Hamamatsu C9100-50 camera (Hamamatsu, Japan). FM4-64 was excited at 561 nm and imaged using a 705W90 nm band pass filter. Lucifer yellow was exited at 406 nm and imaged using a 527W55 nm band pass filter. Calcofluor white was excited at 406 nm and imaged using a 445W60 nm band pass filter. GFP and fluorescein were excited at 488 nm and imaged using a 527W55 band pass filter. Images were processed with ImageJ and Adobe Photoshop software.

### Vacuole staining with FM4-64

Cells were grown overnight to early logarithmic phase in YPD and inoculated in YPD or YPD/EDTA. 0.25 OD units were stained with 10 μM FM4-64 (in dimethyl sulfoxide) in 1 ml of the respective medium for 30 min at 30 °C. Cells were washed twice, chased for 45 min at 30 °C and analyzed with a confocal microscope.

### Lucifer yellow internalization assay

A total of 0.25 OD_600__nm_ units were removed from the culture, pelleted and resuspended in 90 μl of the respective medium. Lucifer yellow was added from a 10× stock solution to a final concentration of 2.3 mM. Cells were incubated for 75 min at 30 °C before the dye was removed by 5 washes with 1× PBS. Fluorescence was acquired on a FACSCanto flow cytometer (Becton Dickinson) and analyzed using FlowJo software (Tree Star). Lucifer yellow was excited with the 403 nm laser and detected in the AmCyan channel (BP 525/20).

### Macrophage infection

Bone marrow cells were obtained by flushing the femurs and tibias of naive female C57BL/6 mice (Harlan). The extracted cells were differentiated into bone marrow-derived macrophages (BMDM) for 5–7 days using complete DMEM supplemented with L929 conditioned media at 37 °C^78^. Differentiated BMDM were cultured in DMEM medium supplemented with 1% penicillin/streptomycin, 10% FCS and 10 mM HEPES at 37 °C and 5% CO_2_ for 2–12 h before being infected with late stationary phase promastigotes at a ratio of 1:5 (macrophage: *Leishmania*). Macrophages were incubated with parasites for 6 h at 37 °C on cell culture coverslips (Thermomanx) in 24-well plates (500’000 macrophages per well). Non-phagocytosed parasites were removed by 2 washes with 1× PBS and fresh DMEM medium was added to the wells. Cells were fixed after 24, 48, and 72 h with 4% PFA for 15–20 min and stained with Diff-Quik (Dade Behring). Phase contrast pictures were taken with a Leica microscope and the number of amastigotes quantified in >100 macrophages per cell line.

### Data availability

All source data are available from the corresponding author upon request.

## Electronic supplementary material

Supplementary Information

## References

[1] Boynton PJ, Greig D (2014). The ecology and evolution of non-domesticated Saccharomyces species. Yeast.

[2] Scalbert A (1991). Antimicrobial properties of tannins. Phytochemistry.

[3] Cos P (2004). Proanthocyanidins in health care: current and new trends. Curr. Med. Chem..

[4] Rieuwerts JS, Thornton I, Farago ME, Ashmore MR (2015). Factors influencing metal bioavailability in soils: preliminary investigations for the development of a critical loads approach for metals. Chem. Spec. Bioavailab..

[5] González-Guerrero M, Escudero V, Saéz Aacute, Tejada-Jiménez M (2016). Transition metal transport in plants and associated endosymbionts: Arbuscular Mycorrhizal fungi and rhizobia. Front. Plant Sci..

[6] Lesuisse E, Simon-Casteras M, Labbe P (1998). Siderophore-mediated iron uptake in Saccharomyces cerevisiae: the SIT1 gene encodes a ferrioxamine B permease that belongs to the major facilitator superfamily. Microbiology.

[7] Heymann P, Ernst JF, Winkelmann G (1999). Identification of a fungal triacetylfusarinine C siderophore transport gene (TAF1) in Saccharomyces cerevisiae as a member of the major facilitator superfamily. Biometals.

[8] Heymann P, Ernst JF, Winkelmann G (2000). A gene of the major facilitator superfamily encodes a transporter for enterobactin (Enb1p) in Saccharomyces cerevisiae. Biometals.

[9] Yun CW, Tiedeman JS, Moore RE, Philpott CC (2000). Siderophore-iron uptake in saccharomyces cerevisiae. Identification of ferrichrome and fusarinine transporters. J. Biol. Chem..

[10] Yun CW (2000). Desferrioxamine-mediated Iron Uptake in Saccharomyces cerevisiae. J. Biol. Chem..

[11] Citiulo F (2012). Candida albicans scavenges host zinc via Pra1 during endothelial invasion. PLoS Pathog..

[12] Moore RE, Kim Y, Philpott CC (2003). The mechanism of ferrichrome transport through Arn1p and its metabolism in Saccharomyces cerevisiae. PNAS.

[13] Froissard M (2007). Trafficking of siderophore transporters in saccharomyces cerevisiae and intracellular fate of ferrioxamine B conjugates. Traffic.

[14] Weissman Z, Shemer R, Conibear E, Kornitzer D (2008). An endocytic mechanism for haemoglobin-iron acquisition in Candida albicans. Mol. Microbiol..

[15] Patel N, Singh SB, Basu SK, Mukhopadhyay A (2008). Leishmania requires Rab7-mediated degradation of endocytosed hemoglobin for their growth. Proc. Natl Acad. Sci. USA.

[16] Li L, Chen OS, McVey Ward D, Kaplan J (2001). CCC1 is a transporter that mediates vacuolar iron storage in yeast. J. Biol. Chem..

[17] MacDiarmid CW, Gaither LA, Eide D (2000). Zinc transporters that regulate vacuolar zinc storage in Saccharomyces cerevisiae. EMBO J..

[18] Rees EM, Lee J, Thiele DJ (2004). Mobilization of intracellular copper stores by the ctr2 vacuolar copper transporter. J. Biol. Chem..

[19] Blaby-Haas CE, Merchant SS (2014). Lysosome-related organelles as mediators of metal homeostasis. J. Biol. Chem..

[20] Docampo R, de Souza W, Miranda K, Rohloff P, Moreno SNJ (2005). Acidocalcisomes - conserved from bacteria to man. Nat. Rev. Microbiol..

[21] Docampo R, Moreno SNJ (2011). Acidocalcisomes. Cell. Calcium.

[22] Van Wazer JR, Callis CF (1958). Metal complexing by phosphates. Chem. Rev..

[23] Momeni A, Filiaggi MJ (2014). Comprehensive study of the chelation and coacervation of alkaline earth metals in the presence of sodium polyphosphate solution. Langmuir.

[24] Cramer CL, Davis RH (1984). Polyphosphate-cation interaction in the amino acid-containing vacuole of Neurospora crassa. J. Biol. Chem..

[25] Kim KS, Rao NN, Fraley CD, Kornberg A (2002). Inorganic polyphosphate is essential for long-term survival and virulence factors in Shigella and Salmonella spp. PNAS.

[26] Lander N, Ulrich PN, Docampo R (2013). Trypanosoma brucei vacuolar transporter chaperone 4 (TbVtc4) is an acidocalcisome polyphosphate kinase required for in vivo infection. J. Biol. Chem..

[27] Smith SA (2006). Polyphosphate modulates blood coagulation and fibrinolysis. PNAS.

[28] Hothorn M (2009). Catalytic core of a membrane-associated eukaryotic polyphosphate polymerase. Science.

[29] Desfougères Y, Gerasimaitė RU, Jessen HJ, Mayer A (2016). Vtc5, a novel subunit of the vacuolar transporter chaperone complex, regulates polyphosphate synthesis and phosphate homeostasis in yeast. J. Biol. Chem..

[30] Ruotolo R, Marchini G, Ottonello S (2008). Membrane transporters and protein traffic networks differentially affecting metal tolerance: a genomic phenotyping study in yeast. Genome Biol..

[31] Chesi A, Kilaru A, Fang X, Cooper AA, Gitler AD (2012). The role of the parkinson’s disease gene PARK9 in essential cellular pathways and the manganese homeostasis network in yeast. PLoS ONE.

[32] Rosenfeld L (2010). The effect of phosphate accumulation on metal ion homeostasis in Saccharomyces cerevisiae. J. Biol. Inorg. Chem..

[33] Uttenweiler A, Schwarz H, Neumann H, Mayer A (2007). The vacuolar transporter chaperone (VTC) complex is required for microautophagy. Mol. Biol. Cell..

[34] Saito K, Ohtomo R, Kuga-Uetake Y, Aono T, Saito M (2005). Direct labeling of polyphosphate at the ultrastructural level in Saccharomyces cerevisiae by using the affinity of the polyphosphate binding domain of Escherichia coli exopolyphosphatase. Appl. Environ. Microbiol..

[35] Werner TP, Amrhein N, Freimoser FM (2007). Specific localization of inorganic polyphosphate (poly P) in fungal cell walls by selective extraction and immunohistochemistry. Fungal. Genet. Biol..

[36] Urech K, Dürr M, Boller T, Wiemken A, Schwencke J (1978). Localization of polyphosphate in vacuoles of Saccharomyces-Cerevisiae. Arch. Microbiol..

[37] Tijssen JP, Beekes HW, Van Steveninck J (1982). Localization of polyphosphates in Saccharomyces fragilis, as revealed by 4’,6-diamidino-2-phenylindole fluorescence. Biochim. Biophys. Acta..

[38] Gerasimaite R, Sharma S, Desfougères Y, Schmidt A, Mayer A (2014). Coupled synthesis and translocation restrains polyphosphate to acidocalcisome-like vacuoles and prevents its toxicity. J. Cell. Sci..

[39] Ogawa N, DeRisi J, Brown PO (2000). New components of a system for phosphate accumulation and polyphosphate metabolism in Saccharomyces cerevisiae revealed by genomic expression analysis. Mol. Biol. Cell..

[40] Avraham N, Soifer I, Carmi M, Barkai N (2013). Increasing population growth by asymmetric segregation of a limiting resource during cell division. Mol. Syst. Biol..

[41] Sethuraman A, Rao NN, Kornberg A (2001). The endopolyphosphatase gene: essential in Saccharomyces cerevisiae. PNAS.

[42] Wiesenberger G (2007). Mg2+ deprivation elicits rapid Ca2+ uptake and activates Ca2+ /calcineurin signaling in Saccharomyces cerevisiae. Eukaryot. Cell..

[43] MacDiarmid CW, Gardner RC (1998). Overexpression of the Saccharomyces cerevisiae magnesium transport system confers resistance to aluminum ion. J. Biol. Chem..

[44] Graschopf A (2001). The yeast plasma membrane protein Alr1 controls Mg2+ homeostasis and is subject to Mg2+ -dependent control of its synthesis and degradation. J. Biol. Chem..

[45] Bui DM, Gregan J, Jarosch E, Ragnini A, Schweyen RJ (1999). The bacterial magnesium transporter CorA can functionally substitute for its putative homologue Mrs2p in the yeast inner mitochondrial membrane. J. Biol. Chem..

[46] Gregan J (2001). The mitochondrial inner membrane protein Lpe10p, a homologue of Mrs2p, is essential for magnesium homeostasis and group II intron splicing in yeast. Mol. Gen. Genet..

[47] Cui Y (2015). A novel mitochondrial carrier protein Mme1 acts as a yeast mitochondrial magnesium exporter. Biochim. Biophys. Acta..

[48] Pisat NP, Pandey A, MacDiarmid CW (2009). MNR2 regulates intracellular magnesium storage in Saccharomyces cerevisiae. Genetics.

[49] Wiederkehr A, Meier KD, Riezman H (2001). Identification and characterization of Saccharomyces cerevisiae mutants defective in fluid‐phase endocytosis. Yeast.

[50] Seaman MN, McCaffery JM, Emr SD (1998). A membrane coat complex essential for endosome-to-Golgi retrograde transport in yeast. J. Cell. Biol..

[51] Balderhaar HJK (2010). The Rab GTPase Ypt7 is linked to retromer-mediated receptor recycling and fusion at the yeast late endosome. J. Cell. Sci..

[52] Arlt H, Reggiori F, Ungermann C (2015). Retromer and the dynamin Vps1 cooperate in the retrieval of transmembrane proteins from vacuoles. J. Cell. Sci..

[53] Garcia-del Portillo F, Foster JW, Maguire ME, Finlay BB (1992). Characterization of the micro-environment of Salmonella typhimurium-containing vacuoles within MDCK epithelial cells. Mol. Microbiol..

[54] Eriksson S, Lucchini S, Thompson A, Rhen M, Hinton J (2003). Unravelling the biology of macrophage infection by gene expression profiling of intracellular Salmonella enterica. Mol. Microbiol..

[55] Mann FM, VanderVen BC, Peters RJ (2011). Magnesium depletion triggers production of an immune modulating diterpenoid in Mycobacterium tuberculosis. Mol. Microbiol..

[56] Lavigne JP, O’callaghan D, Blanc-Potard AB (2005). Requirement of MgtC for Brucella suis intramacrophage growth: a potential mechanism shared by Salmonella enterica and Mycobacterium tuberculosis for adaptation to a low-Mg2+ environment. Infect. Immun..

[57] Docampo R, Huang G (2016). Acidocalcisomes of eukaryotes. Curr. Opin. Cell. Biol..

[58] Antoine JC, Prina E, Lang T, Courret N (1998). The biogenesis and properties of the parasitophorous vacuoles that harbour Leishmania in murine macrophages. Trends Microbiol..

[59] Lanza H (2004). Comparative effect of ion calcium and magnesium in the activation and infection of the murine macrophage by Leishmania major. Biol. Res..

[60] Misslitz A, Mottram JC, Overath P, Aebischer T (2000). Targeted integration into a rRNA locus results in uniform and high level expression of transgenes in Leishmania amastigotes. Mol. Biochem. Parasit..

[61] Haas H (2003). Molecular genetics of fungal siderophore biosynthesis and uptake: the role of siderophores in iron uptake and storage. Appl. Microbiol. Biotechnol..

[62] Nguyen MK, Kurtz I (2006). Quantitative interrelationship between Gibbs-Donnan equilibrium, osmolality of body fluid compartments, and plasma water sodium concentration. J. Appl. Physiol..

[63] Kumble KD, Kornberg A (1996). Endopolyphosphatases for long chain inorganic polyphosphate in yeast and mammals. J. Biol. Chem..

[64] Gerasimaite R, Mayer A (2017). Ppn2, a novel Zn(2+)-dependent polyphosphatase in the acidocalcisome-like yeast vacuole. J. Cell. Sci..

[65] Wiemken A, Dürr M (1974). Characterization of amino acid pools in the vacuolar compartment of Saccharomyces cerevisiae. Arch. Microbiol..

[66] Tomitori H (2001). Multiple polyamine transport systems on the vacuolar membrane in yeast. Biochem. J..

[67] Cini N, Ball V (2014). Polyphosphates as inorganic polyelectrolytes interacting with oppositely charged ions, polymers and deposited on surfaces: fundamentals and applications. Adv. Colloid. Interface. Sci..

[68] Coppens I, Baudhuin P, Opperdoes FR, Courtoy PJ (1993). Role of acidic compartments in Trypanosoma brucei, with special reference to low-density lipoprotein processing. Mol. Biochem. Parasit..

[69] Scott DA, Docampo R, Dvorak JA, Shi S, Leapman RD (1997). In situ compositional analysis of acidocalcisomes in Trypanosoma cruzi. J. Biol. Chem..

[70] Mullin KA (2001). Regulated degradation of an endoplasmic reticulum membrane protein in a tubular lysosome in Leishmania mexicana. Mol. Biol. Cell..

[71] Huang G (2011). Adaptor protein-3 (AP-3) complex mediates the biogenesis of acidocalcisomes and is essential for growth and virulence of Trypanosoma brucei. J. Biol. Chem..

[72] Vannier-Santos MA (1999). Impairment of sterol biosynthesis leads to phosphorus and calcium accumulation in Leishmania acidocalcisomes. Microbiology.

[73] Cruz A, Coburn CM, Beverley SM (1991). Double targeted gene replacement for creating null mutants. PNAS.

[74] Price HP (2003). Myristoyl-CoA:protein N-myristoyltransferase, an essential enzyme and potential drug target in kinetoplastid parasites. J. Biol. Chem..

[75] Azevedo C, Livermore T, Saiardi A (2015). Protein polyphosphorylation of lysine residues by inorganic polyphosphate. Mol. Cell..

[76] Desfougères Y, Neumann H, Mayer A (2016). Organelle size control - increasing vacuole content activates SNAREs to augment organelle volume through homotypic fusion. J. Cell. Sci..

[77] Kulakova AN (2011). Direct quantification of inorganic polyphosphate in microbial cells Using 4’-6-Diamidino-2-Phenylindole (DAPI). Environ. Sci. Technol..

[78] Meerpohl HG, Lohmann Matthes ML, Fischer H (1976). Studies on the activation of mouse bone marrow‐derived macrophages by the macrophage cytotoxicity factor (MCF). Eur. J. Immunol..

