# Supplementary material for: Magnesium uptake by connecting fluid-phase endocytosis to an intracellular inorganic cation filter
Source: Nat Commun. 2017 Dec 1;8:1879. doi: 10.1038/s41467-017-01930-5 (PMC5709425; doi:10.1038/s41467-017-01930-5)
Supplement: Supplementary file 1 — Supplementary Information [file 41467_2017_1930_MOESM1_ESM.pdf]

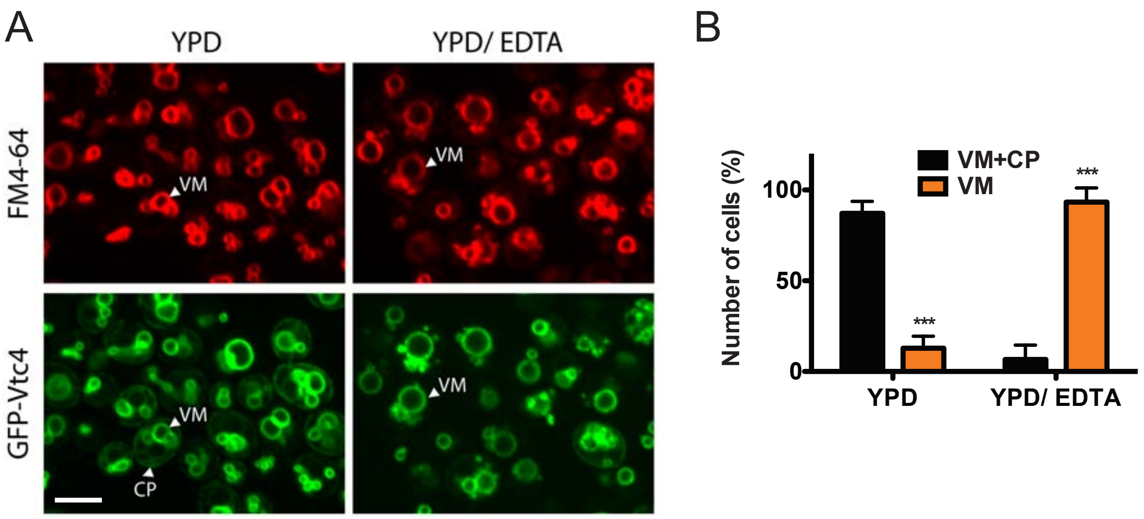

Supplementary Figure 1: Vtc4 localization under metal limitation.

(A) Wildtype cells, expressing genomically integrated GFP-Vtc4, were pre-cultured on YPD to early-logarithmic growth phase and stained with FM4-64. Cells were then transferred into YPD or YPD/EDTA and analyzed by confocal microscopy after 6 hours of growth. VM: vacuolar membrane, CP: cell periphery. Scale bar: 5 μm. **(B)** Quantification of GFP-Vtc4 localization. 60 cells were analysed per condition and experiment. Results represent mean ± SD of three independent experiments. Statistical differences were determined using Student's t-test comparing the localization of GFP-Vtc4 on YPD versus YPD/EDTA; ***p≤0.0005.

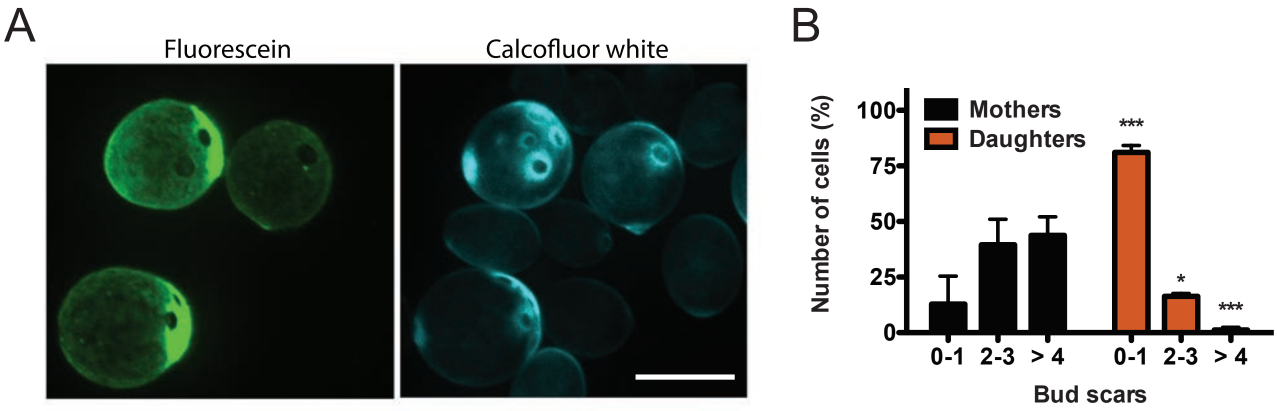

Supplementary Figure 2: Growth of mother and daughter cells on YPD/EDTA.
Wildtype cells were pre-cultured on YPD. The cell walls of the inoculum were chemically cross-linked to sulfo-NHS-LC-LC-biotin and then stained with fluorescein-conjugated avidin. After washing and cultivation for 16 hours on YPD/EDTA, the cells were collected. Bud scars were stained with Calcofluor white and analyzed by confocal microscopy. **(A)** Maximum projection of z-stacks of 30 optical sections of biotinylated mother cells stained with fluorescein-avidin, taken at 0.3 μm intervals. Scale bar: 5 μm. **(B)** Quantification of A. Results represent mean ± SD of three independent experiments. 250 cells were analysed per experiment. Statistical differences were determined using Student's t-test comparing the number of bud scars between mother and daughter populations; *$p \leq 0.05$, ***$p \leq 0.001$.

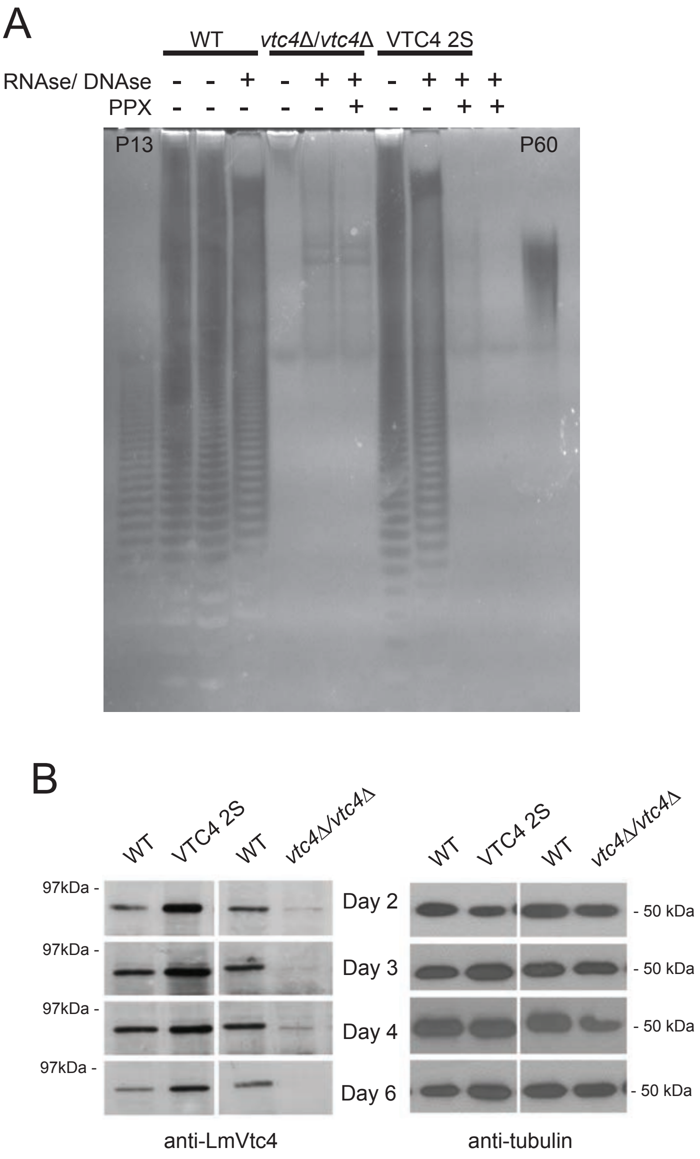

Supplementary Figure 3: Characterization of *Leishmania major* mutants

(A) Gel analysis of polyP levels. Promastigotes of wildtype, a double-knockout of LmVTC4 (*vtc4*Δ/*vtc4*Δ) and of the double-knockout reconstituted with a wildtype allele of LmVTC4 (VTC4 2S) were inoculated in M199 medium. PolyP was extracted from logarithmically grown cells and analyzed by 35% gel electrophoresis and DAPI staining. Samples were treated with RNAse/DNAse and the polyphosphatase Ppx1 as indicated. (B) Vtc4 abundance at different growth phases. At the indicated times after inoculation in M199 medium, aliquots were withdrawn and cells lysed by 5 successive freeze/ thaw cycles in the presence of protease inhibitors. Protein extracts were analyzed by SDS-PAGE and Western blotting against LmVtc4 or tubulin. 20 μg protein were loaded per lane.

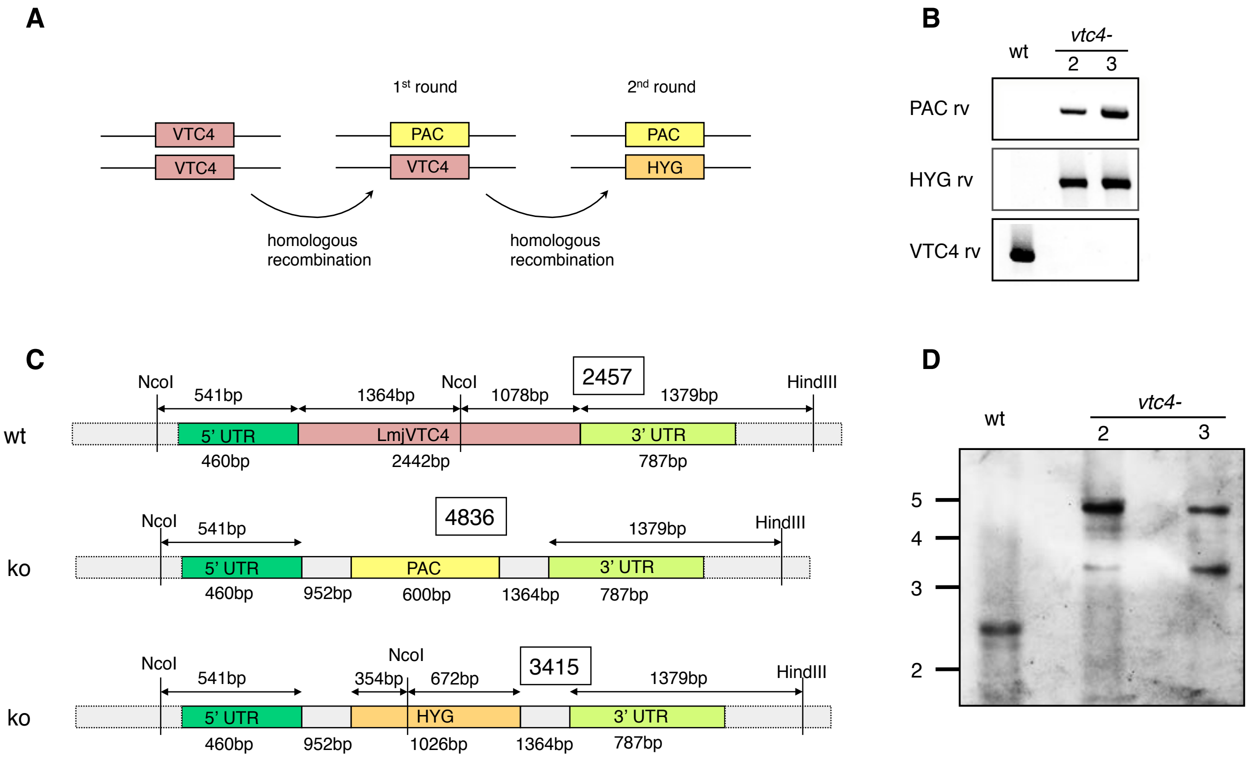

Supplementary Fig. 4: *VTC4* knock-out in *L. major*

(A) The two alleles of the single copy LmVTC4 gene were replaced by drug resistance genes in two subsequent rounds of homologous recombination. Gene replacement was verified **(B)** by PCR, employing the same forward primer with reverse primers annealing in the respective genes, and **(C-D)** by southern blot. Genomic DNA was digested by NcoI and HindIII, and fragments were hybridized with a 3'UTR specific probe. Restriction sites and expected sizes of the labeled fragments are displayed in C. Each allelic combination gives rise to a fragment of a different size, thereby showing one band for wt and two bands for *vtc4-* clones. PAC, puromycin-resistance gene; HYG, hygromycin-resistance gene; UTR, untranslated region.

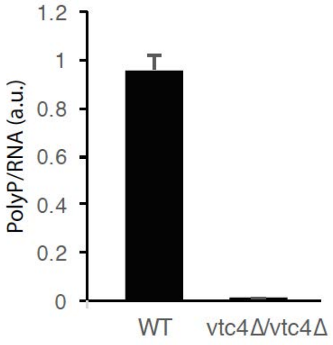

Supplementary Fig. 5: PolyP content of *Leishmania*, measured by enzymatic degradation

PolyP levels during promastigote growth. Promastigotes of wildtype and a double-knockout of LmVTC4 (*vtc4Δ vtc4Δ*) were inoculated in M199 medium. In logarithmic phase, they were analyzed for their polyP content. PolyP was isolated through phenol/chloroform extraction and degraded by incubation with purified yeast Ppx1. Released orthophosphate was quantified by malachite green. Results represent mean ± SD of three independent experiments.

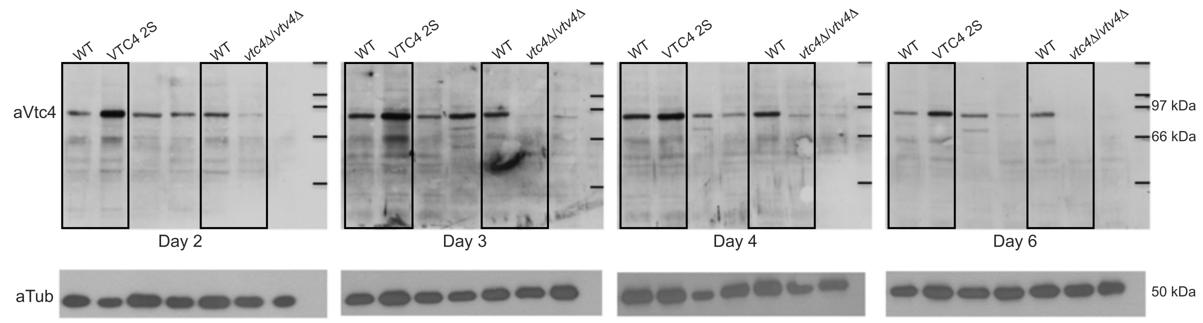

Supplementary Fig. 6: Non-cropped scans of blots in SFig. 3B.

Lanes used in that figure are surrounded by a black rectangle.

Supplementary Table 1: Yeast strains

Strain	Genotype	Reference/ Source
BY4741	MATa his3Δ1 leu2Δ0 met15Δ0 ura3Δ0	Thermo Scientific
BY4741 *vtc4Δ*	BY4741 vtc4::kanMX	Euroscarf
BY4741 *vtc1Δ*	BY4741 vtc1::kanMX	Euroscarf
BY4741 *vtc3Δ*	BY4741 vtc3::kanMX	Euroscarf
BY4742 Vtc4^{R264A}	MATα his3Δ1 leu2Δ0 lys2Δ0 ura3Δ0 vtc4::kanMX P_{VTC4}VTC4^{R264A}(HIS3)	Hothorn, 2009
BY4742 GFP-VTC4	MATα his3Δ1 leu2Δ0 lys2Δ0 ura3Δ0 P_{ADH}GFP-VTC4 (natNT2)	Uttenweiler, 2007
DY1457	MATα ade6 can1 his3 leu2 trp1 ura3	D. Eide, Wisconsin
DY1457 *alr1Δ*	DY1457 alr1::HIS3	D. Eide, Wisconsin
BY4741 *mnr2Δ*	BY4741 mnr2::kanMX	Euroscarf
BY4741 *alr2Δ*	BY4741 alr2::kanMX	Euroscarf
BY4741 *mrs2Δ*	BY4741 mrs2::kanMX	Euroscarf
BY4741 *mfm1Δ*	BY4741 mfm1::kanMX	Euroscarf
BY4741 *mme1Δ*	BY4741 mme1::kanMX	Euroscarf
DTY165 *ctr2Δ*	*MATα ura3-52 his6 leu2-3,-112 his3-Δ200 trp1-901 lys2-801 suc2-Δ* ctr2::HIS3	Rees, 2004
BY4741 *fth1Δ*	BY4741 fth1::kanMX	Euroscarf
BY4741 *smf3Δ*	BY4741 smf3::kanMX	Euroscarf
BY4741 *yvc1Δ*	BY4741 yvc1::kanMX	Euroscarf
BY4741 *zrt3Δ*	BY4741 zrt3::kanMX	Euroscarf
BY4741 *vps4Δ*	BY4741 vps4::kanMX	Euroscarf
BY4741 *atg8Δ*	BY4741 atg8::kanMX	Euroscarf
BY4741 *sac6Δ*	BY4741 sac6::kanMX	Euroscarf
BY4741 *vrp1Δ*	BY4741 vrp1::kanMX	Euroscarf
BY4741 *end3Δ*	BY4741 end3::kanMX	Euroscarf
BY4741 *sla1Δ*	BY4741 sla1::kanMX	Euroscarf
BY4741 *arc18Δ*	BY4741 arc18::kanMX	Euroscarf
BY4741 *vps26Δ*	BY4741 vps26::kanMX	Euroscarf
BY4741 *vps29Δ*	BY4741 vps29::kanMX	Euroscarf
BY4741 *vps35Δ*	BY4741 vps35::kanMX	Euroscarf

Supplementary table 2: Oligonucleotides used in this study

Name	Sequence (5’ --> 3’)	Purpose
AS12	GCCCATTTATACCCATAT	KO check Kan rv
AS267	GAGCTGTCGCAAACAAGTTCG	KO check VTC4 fw
YD012	TATCGCTTGTTACGGTCGGT	KO check VTC1 fw
AS235	TCCAAGTAGTACCTCCTTAGCGC	KO check VTC3 fw
SK114	CATTTTCTGGTTTGGTAGTCC	KO check MNR2 fw
SK115	GACGGTAGAGACTACTATTGC	KO check ALR2 fw
SK116	GATTGATCGACCAGCAGCTTG	KO check MRS2 fw
SK117	CAATGTAGTAGCCACCTCA	KO check FTH1 fw
SK118	CTGATTCTGTGAGGTTGC	KO check SMF3 fw
SK119	CACTGTTGCTATGTCGGAG	KO check YVC1 fw
SK120	GAGATGATTTCACCTACTGG	KO check ZRT3 fw
SK121	CACAACACGTCATGGTTGTTC	KO check VPS4 fw
SK122	CCGTGAAATCATAGCACATG	KO check ATG8 fw
SK123	GATCCTGACCGGATATAGGGTC	KO check SAC6 fw
SK124	CATAACCTAGTCACTGCTTACG	KO check VRP1 fw
SK125	GACGATCGTGTAGGACCC	KO check END3 fw
SK126	CTCTCAGTTTCACAGACTCG	KO check ARC18 fw
SK127	CGAAATCGAGATACATCAGAC	KO check SLA1 fw
**vps26 fw**	TCGACAAACCCGTATAAGAC	KO check VPS26 fw
**vps29 fw**	TTAACCGTAGCATCATTGTG	KO check VPS29 fw
**vps35 fw**	AAGATTGTTGATTGGATTGC	KO check VPS35 fw
LmjVtc4_5’up fw (HindIII)	CACAAGCTTAGCGAAGCCAAGCCGCTC	5’UTR into pX63 KO cassette
LmjVtc4_5’lo rv (SalI)	CTAGTCGACGATGGTGGCGGGGCGCC	5’UTR into pX63 KO cassette
LmjVtc4_3’up fw (SmaI)	CATCCCGGGCGGTGCGCGTGCGTTGC	3’UTR into pX63 KO cassette
LmjVtc4_3’lo fw (BglII)	GACAGATCTGAGGGAGCGGTTCGGTTC	3’UTR into pX63 KO cassette
fw-ClaILmjVtc4st	CATATCGATATGCCGTACAGCAAGGCATGG	LmVtc4 into pSSU vector
Rv-XmaI-LmjVtc4en	CATCCCGGGCTAGAACGAGTCGCTGCCTGT	LmVtc4 into pSSU vector
NEO fw (SpeI)	CATATCGATATGCCGTACAGCAAGGCATGG	NEO into pSSU vector

NEO rv (XbaI)	CATATCGATATGCCGTACAGCAAGGCATGG	NEO into pSSU vector
IRcpbXmaI-sense	GGACCCGGGTGTGTGCCCTTGTGTGCGT	CPB IR into pSSU vector
IRcpbSpeI-anti	GGCACTAGTTCTAGTCGCGGACGCGG	CPB IR into pSSU vector
k.o. genomic fw	GAGTTCTATCCCCTGCTCATGCT	PCR check Lm Δvtc4/vtc4
k.o. Vtc4.90 rv	GAGGATGTCCTTGGTTGCCTTGT	PCR check Lm Δvtc4/vtc4
k.o. PAC.151 rv	CTCAGTTCTTGCAGCTCGGTGAC	PCR check Lm Δvtc4/vtc4
k.o. Hyg.2 rv	GGTGAGTTCAGGCTTTTTCA	PCR check Lm Δvtc4/vtc4

Supplementary Table 3: Element composition of YPD and metal-depleted YPD, determined by ICP-MS. Means +/- SD from three independent measurements are given.

Metal	YPD (μM)	YPD/ Chelex (μM)
Mg	167.5 ± 8.3	7.3 ± 5.2
Fe	10.9 ± 0.01	2.5 ± 0.05
Mn	0.67 ± 0.3	< 0.18
Zn	19.6 ± 1.3	0.8 ± 0.4
Ca	253.3 ± 1.8	10.5 ± 0.01
Cu	0.75 ± 0.05	0.66 ± 0.06
